# Supplementary material for: Comprehensive Annotation and Functional Exploration of MicroRNAs in Lettuce
Source: Front Plant Sci. 2021 Dec 24;12:781836. doi: 10.3389/fpls.2021.781836 (PMC8739914; doi:10.3389/fpls.2021.781836)
Supplement: Supplementary file 3 [file Data_Sheet_9.ZIP › GSM2230753.plot/Lsa-miRN55_Lsat_1_v5_gn_3_126721.1_2398_TPlot.pdf]

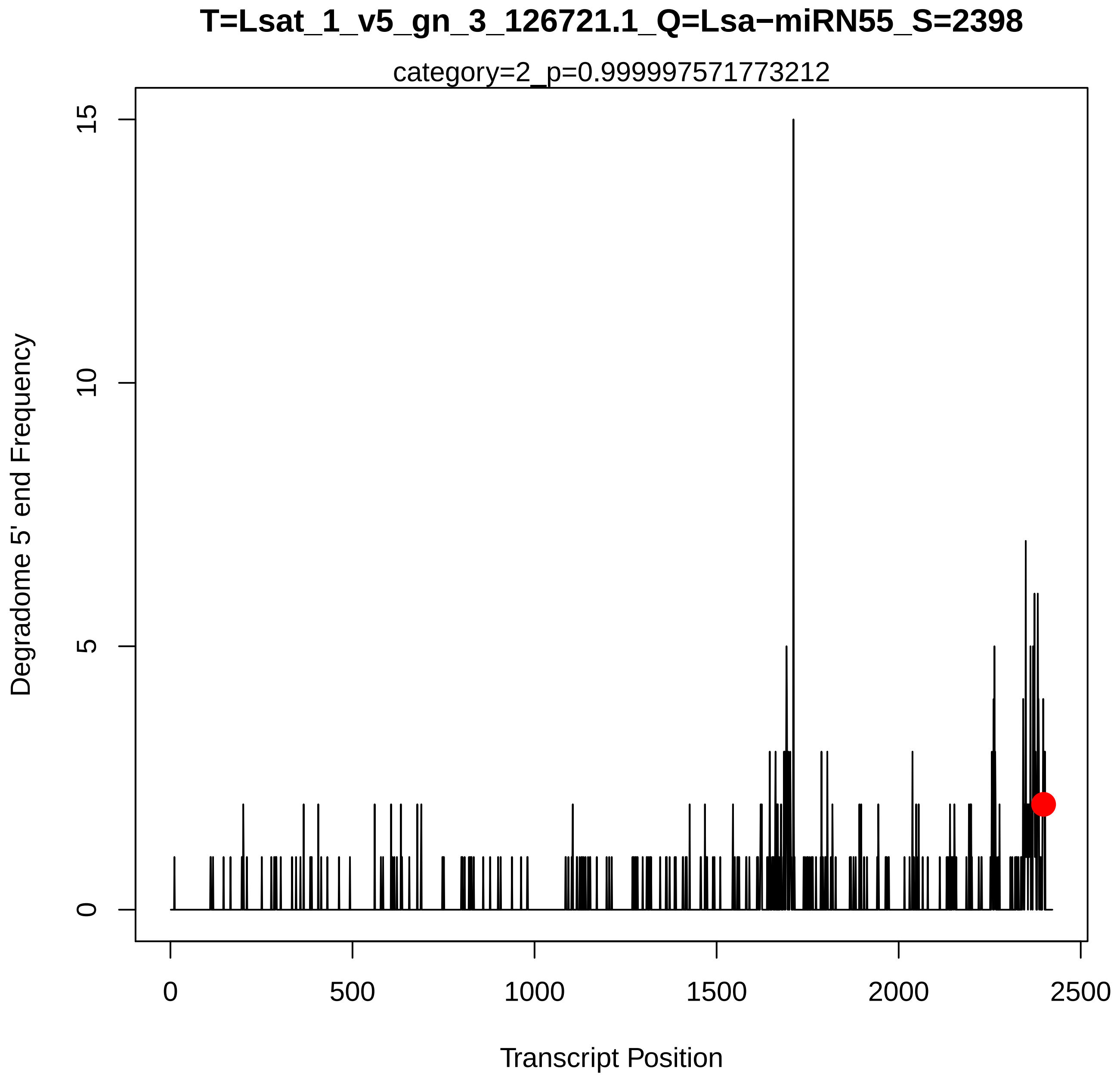

T=Lsat_1_v5_gn_3_126721.1_Q=Lsa−miRN55_S=2398
category=2_p=0.999997571773212
Degradome 5' end Frequency
0
5
10
15
0
500
1000
1500
2000
2500
Transcript Position
